# GABA-Alleviated Oxidative Injury Induced by Salinity, Osmotic Stress and their Combination by Regulating Cellular and Molecular Signals in Rice

**DOI:** 10.3390/ijms20225709

**Published:** 2019-11-14

**Authors:** Mohamed S. Sheteiwy, Hongbo Shao, Weicong Qi, Yousef Alhaj Hamoud, Hiba Shaghaleh, Nasr Ullah Khan, Ruiping Yang, Boping Tang

**Affiliations:** 1 Salt-Soil Agricultural Center, Key Laboratory of Agricultural Environment in the Lower Reaches of Yangtze River Plain, Institute of Agriculture Resources and Environment, Jiangsu Academy of Agricultural Sciences (JAAS), Nanjing 210014, China; salahco_2010@mans.edu.eg (M.S.S.); weicong_qi@126.com (W.Q.); yousef@sina.com (Y.A.H.); khan@hotmail.com (N.U.K.); 2 Department of Agronomy, Faculty of Agriculture, Mansoura University, Mansoura 35516, Egypt; 3 Jiangsu Key Laboratory for Bioresources of Saline Soils, Jiangsu Synthetic Innovation Center for Coastal Bio-agriculture, Yancheng Teachers University, Yancheng 224002, China; yangruiping@ycnu.edu.cn (R.Y.); tangbp@ycnu.edu.cn (B.T.); 4 College of Environment and Safety Engineering, Qingdao University of Science & Technology (QUST), Qingdao 266000, China

**Keywords:** rice, salinity, osmotic stress, combined stress, GABA, phenolic metabolism, *CIPKs* genes

## Abstract

This study was conducted in order to determine the effect of priming with γ-aminobutyric acid (GABA) at 0.5 mM on rice (*Oryza sativa* L.) seed germination under osmotic stress (OS) induced by polyethylene glycol (30 g/L PEG 6000); and salinity stress (S, 150 mM NaCl) and their combination (OS+S). Priming with GABA significantly alleviated the detrimental effects of OS, S and OS+S on seed germination and seedling growth. The photosynthetic system and water relation parameters were improved by GABA under stress. Priming treatment significantly increased the GABA content, sugars, protein, starch and glutathione reductase. GABA priming significantly reduced Na^+^ concentrations, proline, free radical and malonaldehyde and also significantly increased K^+^ concentration under the stress condition. Additionally, the activities of antioxidant enzymes, phenolic metabolism-related enzymes, detoxification-related enzymes and their transcription levels were improved by GABA priming under stress. In the GABA primed-plants, salinity stress alone resulted in an obvious increase in the expression level of Calcineurin B-like Protein-interacting protein Kinases (*CIPKs*) genes such as *OsCIPK01*, *OsCIPK03*, *OsCIPK08* and *OsCIPK15*, and osmotic stress alone resulted in obvious increase in the expression of *OsCIPK02*, *OsCIPK07* and *OsCIPK09*; and OS+S resulted in a significant up-regulation of *OsCIPK12* and *OsCIPK17*. The results showed that salinity, osmotic stresses and their combination induced changes in cell ultra-morphology and cell cycle progression resulting in prolonged cell cycle development duration and inhibitory effects on rice seedlings growth. Hence, our findings suggested that the high tolerance to OS+S is closely associated with the capability of GABA priming to control the reactive oxygen species (ROS) level by inducing antioxidant enzymes, secondary metabolism and their transcription level. This knowledge provides new evidence for better understanding molecular mechanisms of GABA-regulating salinity and osmotic-combined stress tolerance during rice seed germination and development.

## 1. Introduction

Rice is one of the most important cereal crops that serves as the staple food for almost half of the world’s population. Rice is not a salt-tolerant crop, but is suited for cultivation in affected saline soils due to its highly consumption of fresh water for most of the growing season, which could dilute the salts and increase the availability of essential nutrients such as Fe, Mn, N, and P, which contribute to improving rice growth and yield. The mechanism by which rice can tolerate salinity stress is mainly related to the maintenance of ion homeostasis, predominantly low Na^+^/K^+^ or high K^+^/Na^+^ ratios, through exclusion, compartmentation, and partitioning of Na^+^ [1]. In addition, rice plants can tolerate salt by ion exclusion which mainly involves Na^+^ and Cl^−^ transport processes in roots and prevention of the excess accumulation of Na^+^ and Cl^−^ in leaves [2], as well as osmotic stress tolerance which maintains leaf expansion and stomatal conductance [3]. However, rice productivity is affected by salinity stress, which originates from the accumulation of underground salt and is exacerbated by salt mining, deforestation and irrigation [4]. The tolerance limit of rice to saline conditions may vary among the different growth and developmental stages. In this regards, Zhu et al. [5] reported that rice is more tolerance to salinity during the germination and tillering stages, whereas it seems to be more sensitive during early vegetative and reproductive stages.

Salinity, being an important environmental factor, severely causes a significant reduction in the seed germination, seedling growth and development of rice. It has been reported that more than 800 million hectares of the global cultivated area are severely affected by salt stress [2]. A previous study has showed that rice plants experience osmotic stress in saline soil as a result of reduced osmotic potential of the soil solution, and ultimately reduced water uptake by plants [6]. Under the salinity stress condition, the photosynthetic rate decreased due to stomatal closure and resulted in limited availability of CO_2_ and thus altered carbohydrate content of the leaf [7]. Plants can adapt to these conditions by accumulation of compatible solutes such as proline and starch, which function as osmoprotectants and have a vital role in plant adaptation to osmotic stress through stabilization of the tertiary structure of proteins [2]. Salinity reduces the ability of plants to take up water, which leads to increasing of osmotic substances, and causes inhibition of plant growth rate accompanied by metabolic changes similar to those induced by osmotic stress [8]. This action of salinity can induce osmotic stress, oxidative damage, stomatal closure, inhibition of photosynthesis, and damage of cellular structures, and decreased gas exchange rates [9]. Osmotic stress can cause a significant crop yield loss worldwide. It can reduce the plant productivity and seedling growth [10] by affecting the stomatal closure and photosynthesis process [9]. In the present study, PEG (6000) was used to induce osmotic stress in rice plants, being frequently used to induce osmotic stress in several plant species [11,12]. Moreover, the osmotic stress induced by PEG can reduce the photosynthetic rate and chlorophyll content by inhibiting the electron transport system. 

The feedback regulation of plants to the combination of salinity and osmotic stresses is unique and cannot be directly extrapolated from the response of plants to each of the two stresses applied individually [13]. The physiological response of barley was investigated under combinations of two different abiotic stresses [8]; however, the molecular mechanism of plant adaptation to a combination of two different stresses remains a matter of debate [14]; this adaptation might require conflicting or antagonistic responses [14,15]. As such, plants can be adapted to heat stress by increasing the transpiration rate through opening stomata to recover from the high temperature of their leaves. Nevertheless, when plants are exposed to the combination of heat and osmotic stresses, plants have to close their stomata to reduce water loss under the osmotic stress condition [14].

GABA accumulates rapidly in response to biotic and abiotic stresses [16]. Despite the rapid accumulation of GABA during stresses, the specificity of the response and the specific role of GABA under these conditions are still elusive [17]. The accumulation of GABA in different plant cells under the osmotic stress condition requires a specific response which can act as a signaling molecule by modulating the activity of H^+^-ATPase and regulating stomatal movement [17,18]. This action of GABA accumulation has been observed in plants under different environmental stresses such as osmotic stress, oxygen deficiency, mechanical stimulation, low temperature and pathogen attack [16,19]. As a physiological response under abiotic stress, GABA plays vital roles in plants for maintaining the C/N balance, regulating cytosolic pH and scavenging reactive oxygen species (ROS) [20]. Under salinity stress, exogenous GABA application acts as a signaling molecule and functions in phenolic compound enrichment [21], scavenging of ROS [22] and modulating antioxidant enzyme activities in nitrogen metabolic pathways [23]. Recent studies reported that GABA has been implicated in signaling processes affecting the nitrate-uptake system [24] and guidance of the pollen tube [25]. 

Salinity and osmotic stresses have a significant devastating effect limiting worldwide crop production. Taking into consideration the expected increasing world population and food demand, finding ways to improve crop tolerance to abiotic stress constraints is an urgent issue for further improving agricultural production and enhancing global food security. GABA metabolism could be involved in regulation of plant development under abiotic stress through regulation of C and N metabolism [26]. Therefore, the present study hypothesized that GABA could be involved in rice tolerance to salinity and osmotic stresses and their combination. Moreover, the *CIPKs* genes were frequently expressed and participated in plant growth and development during abiotic stresses such as heat, drought, salinity, and chilling stresses [27]. For this reason, we have investigated the effects of GABA on rice *CIPK* genes in responses to the combination of salinity and osmotic stresses to evaluate the potential usefulness of the stress-responsive *CIPK* genes in genetic improvement of stress tolerance. In addition, the present study hypothesized that high accumulation of secondary metabolites during salinity and osmotic stress could facilitate osmotic adjustment and ultimately increase rice tolerance to the combined stresses. This study was undertaken to elucidate the mechanism by which exogenously supplied GABA is involved in the stresses tolerance, in the context of regulation of Na^+^ and K^+^ balance, the photosynthetic system, antioxidant system, cell cycle development, cellular regulation, and controlling stomatal conductance which ultimately might result in improving rice growth under the stress conditions. The current study could significantly contribute to further understanding the tolerance mechanism induced by priming rice seeds with GABA under salinity and osmotic stresses. 

## 2. Results

### 2.1. Effects of GABA Priming on the Morpho-Physiological Parameters under Salinity, Osmotic Stress and OS+S 

The effects of the salinity, osmotic stress and their combination on the physiological parameters of rice seedlings with or without GABA priming are presented in Table 1. Salinity, osmotic stresses, and their combination caused a significant reduction in the germination percentage, germination energy, root length, shoot length, seedling fresh and dry weight, and seedling vigor index as compared with unstressed seedlings. Both the salinity and OS+S stresses induced a greater reduction than the osmotic stress alone (Table 1). However, priming with 0.5 mM GABA improved the physiological parameters under the salinity, osmotic stress and their combination as compared with unprimed plants. Regardless of the effect of salinity and osmotic stress, priming with 0.5 mM GABA improved the germination percentage, germination energy and vigor index by 1.12%, 5.47% and 8.88%, respectively as compared with the control condition (Table 1). Under the osmotic stress, salinity, and OS+S conditions, priming with 0.5 mM of GABA improved the germination percentage by 4.15%, 4.56% and 6.75%; germination energy by 2.85%, 23.79%, and 25.35%; and vigor index by 14.51%, 16.60% and 33.79%, respectively. Irrespective of the priming treatment, the effects of different stresses were different. The combination of salinity and osmotic stress was the most damaging for rice growth followed by individual salinity and osmotic stress (Table 1).

### 2.2. Effects of GABA Priming on the Photosynthetic and Water Relation Parameters under Salinity, Osmotic Stress and OS+S 

Salinity and osmotic stresses and their combination resulted in a significant reduction in the net photosynthetic (Pn), transpiration rate (Tr), stomatal conductance (Gs), intracellular CO_2_ (Ci), chlorophyll content (SPAD), water potential (Ψw), osmotic potential (Ψs) and relative water content (RWC) as compared with unstressed seedlings (Table 2). Priming with 0.5 mM GABA resulted in the highest Pn, Tr, Gs, SPAD, Ψw, Ψs, RWC and WUE as compared with unprimed seedlings; however, the unprimed seedlings resulted in highest Ci (Table 2). As compared with salinity and osmotic stress, the OS+S resulted in the lowest values of Pn, Tr, Gs, Ψw and RWC (Table 2). Interestingly, osmotic stress alone resulted in the lowest SPAD, while the salinity alone resulted in the lowest Ψs. As compared to the unprimed seeds, priming with 0.5 mM GABA improved the Pn, Tr, Gs, SPAD, Ψw, Ψs, RWC and WUE by 39.90%, 43.06%, 59.40%, 28.52%, 32.20%, 42.85%, 48.05% and 76.72%, respectively (Table 2). Under osmotic stress, salinity and OS+S conditions, priming with 0.5 mM GABA improved Pn by 38.21%, 23.86% and 32.85%; Tr by 44.62%, 54.94% and 50.56%; Gs by 16.57%, 38.96% and 49.56%; Ci by 9.39%, 34.53% and 11.44; SPAD by 27.24%, 16.87% and 16.32%; Ψw by 44.76%, 74.65% and 63.35%; Ψs by 17.64%, 36.48% and 3.84%; RWC by 55.29%, 44.90% and 57.50%; and WUE by 33.85%, 43.26% and 38.35%, respectively, as compared with unprimed seeds (Table 2). The present study suggested that application of 0.5 mM GABA to stressed plants with salinity, osmotic stress and their combination affected photosynthetic mechanisms, such as CO_2_ diffusion through stomatal control. Moreover, it also affected the leaf water balance by controlling water and osmotic potential of the leaf to maintain water uptake for plant growth with relatively little water loss by the plant.

### 2.3. Effects of GABA Priming on the Sugar, Protein, Starch and GABA Contents under Salinity, Osmotic Stress and OS+S 

The mean data concerning effects of salinity and osmotic stress and their combination on the sugars, protein, and starch contents are presented in Table 3. The results reported that osmotic stress, salinity and their combination decreased the sugar content by 49.60%, 60.32%, 66.64%; protein content by 54.85%, 57.16% and 64.51; and starch content by 49.90%, 59.12%, and 56.54%, respectively, as compared with the unstressed condition. However, priming with GABA improved the sugar content under osmotic stress, salinity and OS+S by 36.36%, 44.88% and 59.07%; protein content by 32.28%, 52.26% and 49.56% and starch content by 47.96%, 44.42% and 26.93%, respectively, as compared with unprimed seeds. The osmotic stress, salinity and OS+S resulted in a significant increase in the GABA content as compared with the unstressed condition (Table 3). Priming seeds with GABA significantly improved the GABA content under salinity and osmotic stress and their combination, and the highest GABA content was recorded under osmotic stress as compared with either salinity alone or OS+S stress. The current study emphasized that application of 0.5 mM GABA resulted in the accumulation of sugars, starch and protein (Table 3), which may serve as osmolytes to provide energy and carbon at times when photosynthesis may be inhibited under salinity and osmotic stresses.

### 2.4. Effects of GABA Priming on ROS, MDA, Proline and GR under Salinity, Osmotic Stress and OS+S 

**Figure 1:**
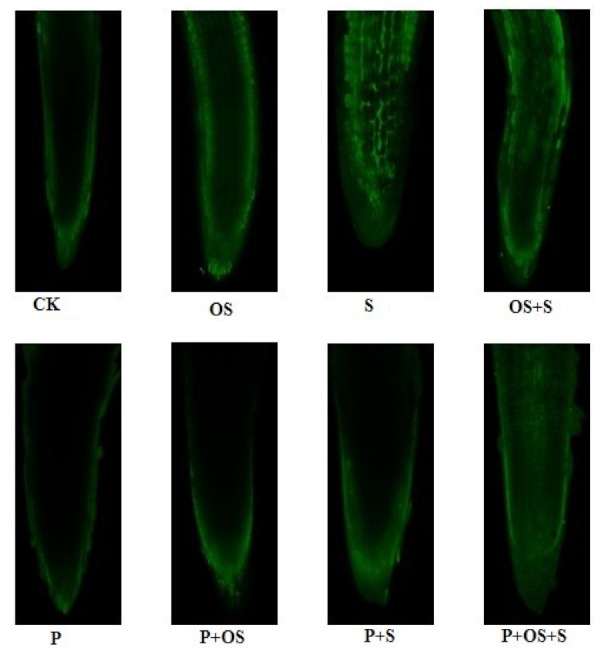
Effects of priming treatment on H_2_O_2_ accumulation in the root cells of rice seedlings exposed to salinity, osmotic stress, and their combined stress (OS+S). P (Priming) OS (Osmotic stress); S (Salinity).

The results revealed that osmotic stress, salinity and OS+S improved ROS, i.e., hydrogen peroxide (H_2_O_2_), superoxide radical (O_2_^−^) and hydroxyl ion (OH^−^) accumulation in the leaf tissues as compared with the control condition (Table 3). Priming treatment significantly reduced the accumulation of the H_2_O_2_ under osmotic stress, salinity and OS+S by 43.75%, 38.01% and 28.91%; O_2_^−^ by 0.37%, 30.88%, and 26.93%; and OH^−^ by 17.64%, 22.38% and 25.64%, respectively, as compared with unprimed seeds (Table 3), and these findings were confirmed by the confocal microscopic investigation as depicted in Figure 1. Plants exposed to osmotic stress, salinity and OS+S experienced a significant increase in proline, malonaldehyde (MDA), and glutathione reductase (GR) concentrations as compared with the unstressed condition (Table 3). Proline, MDA and GR contents were not significantly affected by 0.5 mM GABA priming irrespective of the stress effects. However, the primed seedlings exposed to osmotic stress, salinity and OS+S had the lowest contents of MDA and proline, specifically under osmotic stress. Priming with 0.5 mM GABA resulted in a significant decrease of proline under osmotic stress, salinity, and OS+S, by 28.81%, 43.05% and 35.66%, respectively, relative to unprimed seeds (Table 3). By contrast, priming with 0.5 mM GABA resulted in a significant increase in the GR activity under the stress condition. It could be concluded that priming with 0.5 mM GABA improved GR activity under osmotic stress, salinity and OS+S stresses by 43.41%, 27.8% and 37.46%, respectively, relative to unprimed seeds. It could be stated that 0.5 mM GABA inhibited the release of free radicals by reducing the ROS and MDA levels and thus reducing the cell membrane damage under salinity, osmotic stress and their combination.

### 2.5. Effects of GABA Priming on Ion Accumulation under Salinity, Osmotic Stress and OS+S 

**Figure 2:**
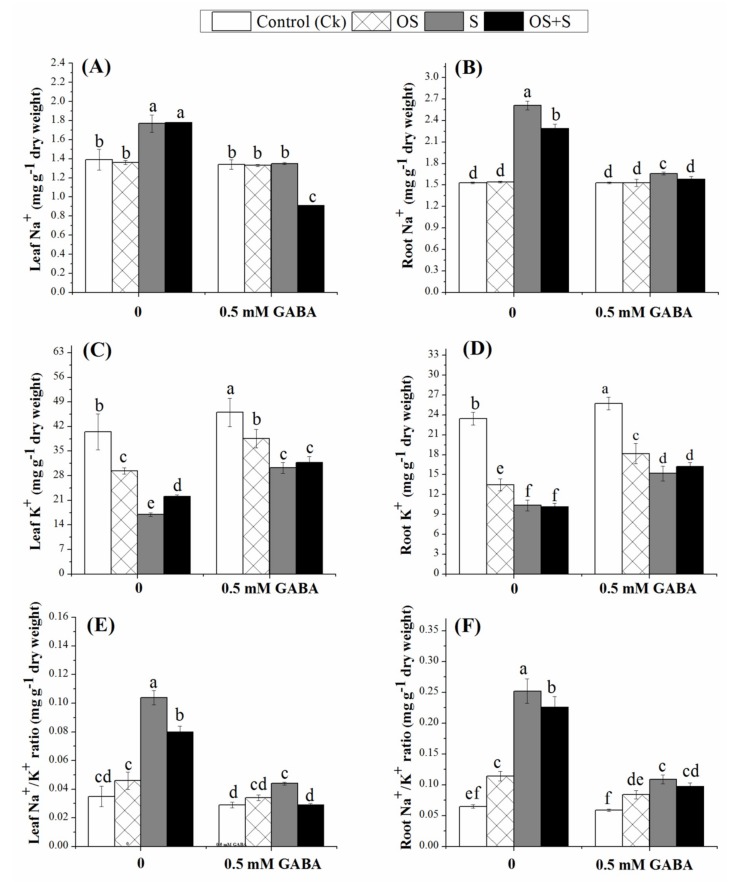
Effects of GABA treatment on the concentration of Na^+^ in the leaf (**A**), Na^+^ in the root (**B**), K^+^ in the leaf (**C**), K^+^ in the root (**D**), Na^+^/K^+^ ratio in the leaf (**E**) and Na^+^/K^+^ ratio in the roots (**F**) of rice seedlings exposed to salinity, osmotic stress, and their combined stress (OS+S). OS (Osmotic stress); S (Salinity).

The obtained results showed that salinity and OS+S stresses significantly increased the Na^+^ concentration in the leaf and root tissues as compared to the control condition, and a higher concentration of Na^+^ was observed in the root as compared to the leaf (Figure 2). Interestingly, the Na^+^ concentration was not significantly reduced by GABA priming in the leaf and root under the unstressed condition. However, under the salinity and OS+S stresses, the GABA priming resulted in a significant reduction of the Na^+^ concentrations in the leaf and root as compared with unprimed seeds. Priming decreased Na^+^ concentrations under osmotic stress, salinity and OS+S stresses by 2.20%, 23.72% and 48.87% in the leaf, and in the root by 0.64%, 36.39% and 31.00%, respectively. Irrespective of the salinity, osmotic stress and their combination, priming with 0.5 mM GABA significantly improved the K^+^ concentration in the leaf and root tissues (Figure 2). It could be concluded that priming seeds with GABA significantly improved the K^+^ concentration in the leaf by 23.84%, 43.89%, and 30.65%, and in the root by 25.96%, 31.68%, and 37.50% under the osmotic stress, salinity and OS+S stresses, respectively (Figure 2). The present study suggested that priming with 0.5 mM GABA has the potential to maintain the balance between the accumulation of Na^+^ in the plant cell and the loss of K^+^ under salinity, osmotic stress and their combination.

### 2.6. Effects of GABA Priming on Enzyme Activities under Salinity, Osmotic Stress and OS+S 

**Figure 3:**
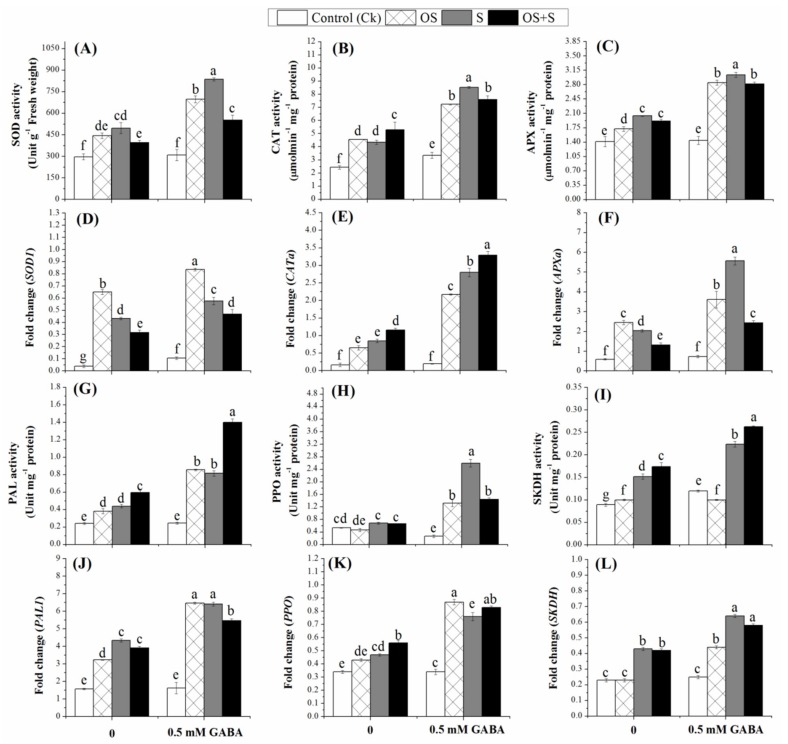
Effects of GABA treatment on SOD (**A**), CAT (**B**), APX (**C**) activities; *SOD1* (**D**); *CATa* (**E**); *APXa* (**F**); PAL (**G**); PPO (**H**); SKDH (**I**); *PAL1* (**J**); *PPO* (**K**) and *SKDH* (**L**) of rice seedlings exposed to salinity, osmotic stress, and their combined stress (OS+S). OS (Osmotic stress); S (Salinity).

As shown in Figure 3A–C, the activities of antioxidant enzymes such as superoxide dismutase (SOD), catalase (CAT) and ascorbic peroxidase (APX) increased in the seedlings exposed to osmotic stress, salinity and OS+S stresses as compared with their respective controls. Priming seeds with 0.5 mM GABA significantly improved the activities of these enzymes under the salinity, osmotic stress and their combination, but the impact was more obvious under the salinity stress as compared with osmotic stress and the combined stress (Figure 3A–C). Compared to the unprimed seeds, SOD activity was improved in the GABA-primed seeds under osmotic stress, salinity and OS+S stresses by 36.46%, 40.64% and 28.29%; CAT activity by 36.92%, 49.06% and 30.17%, and APX activity by 39.64%, 32.89% and 27.65%, respectively (Figure 3A–C). It seems that the application of 0.5 mM GABA can regulate the antioxidant enzymes activity which played a crucial role in scavenging H_2_O_2_ helping to minimize excessive ROS in the stressed plants under salinity, osmotic stress and their combination. 

### 2.7. Effects of GABA Priming on Phenolic Metabolism under Salinity, Osmotic Stress and OS+S 

**Figure 4:**
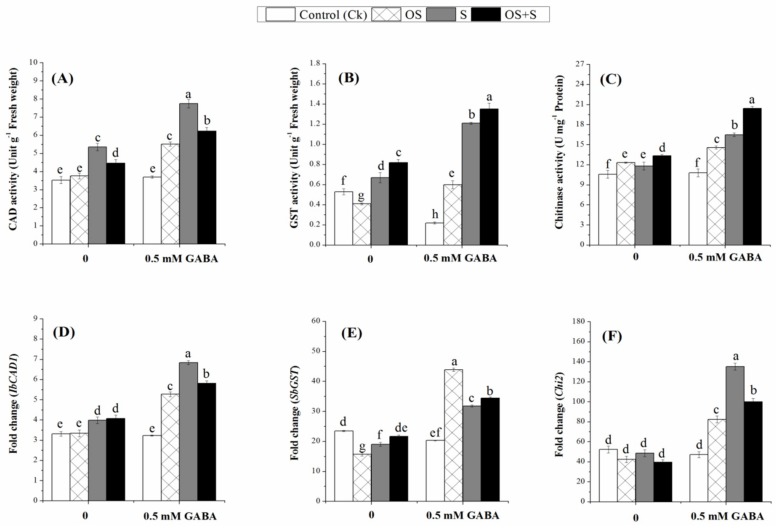
Effects of GABA treatment on the activities of CAD (**A**), GST (**B**) and Chitinase (**C**) and, their transcript levels (**D**–**F**) in rice seedlings exposed to salinity, osmotic stress, and their combined stress (OS+S). OS (Osmotic stress); S (Salinity).

The results presented in Figure 3G–I showed that phenylalanine ammonia-lyase (PAL) activity significantly increased when the seedlings were exposed to the osmotic stress, salinity and OS+S relative to their controls. Priming with 0.5mM GABA resulted in a significant increase in PAL activity as compared to unprimed seeds. The priming treatment resulted in an increase of the PAL activity under osmotic stress, salinity and OS+S stress by 47.3%, 46.07% and 57.42%, respectively, relative to the unprimed seeds (Figure 3G). Similarly, polyphenol oxidase (PPO) activity was increased in the primed seedlings under osmotic stress, salinity and OS+S by 67.72%, 73.73% and 54.16%, respectively (Figure 3H). Interestingly, the PPO activity in the unprimed seedlings under osmotic stress was lower than that in the control seedlings. The results revealed that priming with 0.5 mM GABA resulted in a significant increase in Shikimate dehydrogenase (SKDH) activity under salinity and OS+S, but there was no significant difference under the osmotic stress in primed and unprimed seedlings (Figure 3I). Cinnamyl alcohol dehydrogenase (CAD) activity significantly increased in the primed seedlings exposed to osmotic stress, salinity and OS+S, while there was no significant increase in the CAD activity in the unprimed seeds under osmotic stress (Figure 4A). GABA priming increased the CAD activity under osmotic stress, salinity and OS+S by 31.88%, 30.83% and 28.52%, respectively, relative to unprimed seeds. Hence, in the present study, priming with 0.5 mM GABA resulted in up-regulation of secondary metabolism, such as PAL, PPO and SKDH, which can generate a defense mechanism against oxidative stress induced by salinity, osmotic stress and their combination. 

### 2.8. Effects of GABA Priming on Detoxification-related Enzymes under Salinity, Osmotic Stress and OS+S 

The activities of the detoxification-related enzyme such as Glutathione-s-transferase (GST) and chitinase are shown in Figure 4B,C. Without stress, the GST activity in the primed seedlings was significantly lower than that in the control seedlings (Figure 4B). The GST activity was significantly increased by priming with 0.5 mM GABA under osmotic stress, salinity and OS+S as compared with unprimed seeds. Priming with GABA resulted in an increase in the GST activity under the osmotic stress, salinity and OS+S by 31.66%, 44.62% and 39.25%, respectively, relative to the controls (Figure 4B). Similarly, the chitinase activity was also increased under the osmotic stress, salinity and OS+S as compared to the unstressed condition (Figure 4C). There was no significant difference in the chitinase activity in the primed and unprimed seedlings under the unstressed condition. However, priming with 0.5 mM GABA increased the chitinase activity under the osmotic stress, salinity and OS+S, by 15.54%, 28.34% and 42.09%, respectively, relative to the controls (Figure 4C). 

### 2.9. Effects of GABA Priming on Gene Expression under Salinity, Osmotic Stress and OS+S 

**Figure 5:**
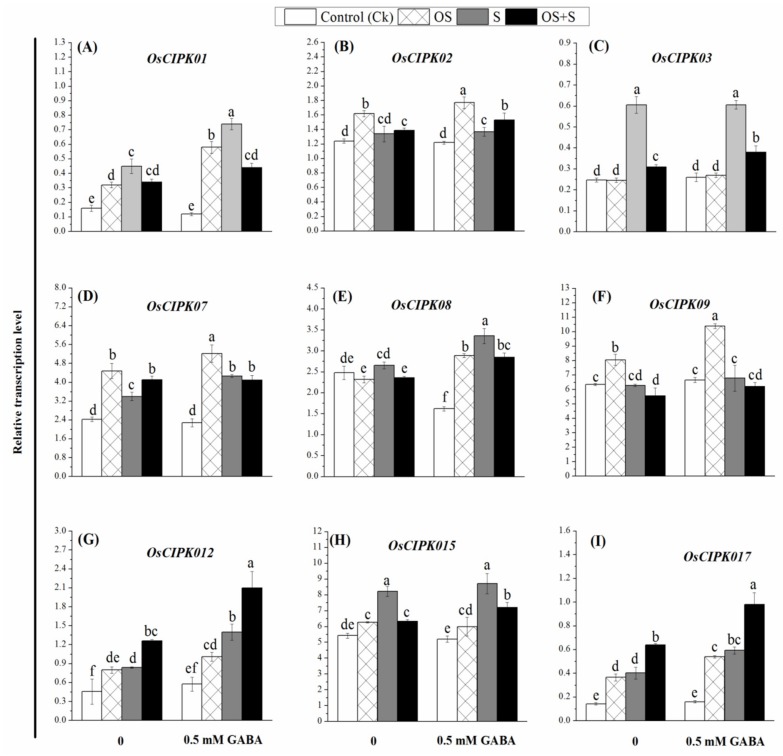
Effects of GABA treatment on the transcription levels of *OsCIPK01* (**A**), *OsCIPK02* (**B**), *OsCIPK03* (**C**), *OsCIPK07* (**D**), *OsCIPK08* (**E**), *OsCIPK09* (**F**), *OsCIPK012* (**G**), *OsCIPK015* (**H**) and *OsCIPK017* (**I**) genes in rice seedlings exposed to salinity, osmotic stress, and their combined stress (OS+S). OS (Osmotic stress); S (Salinity).

In order to further understand the molecular mechanism by which GABA priming could alleviate the detrimental effects of osmotic stress, salinity and their combination, we investigated the transcript levels of *APXa*, *CATa*, *SOD1* (Figure 3D–F)*, PAL1*, *PPO*, *SKDH* (Figure 3J–L), *IbCAD1*, *SbGST*, *Chi2* (Figure 4D–F), *OsCIPK01*, *OsCIPK02*, *OsCIPK03*, *OsCIPK07*, *OsCIPK08*, *OsCIPK09*, *OsCIPK12*, *OsCIPK15*, *OsCIPK17* genes (Figure 5A–I). As shown in Figure 3D–F, *SOD1*, *CATa* and *APXa* were significantly up-regulated in the GABA-primed seedlings under osmotic stress, salinity and OS+S stress relative to their controls. In the primed seedlings, *SOD1* was significantly up-regulated under osmotic stress as compared with salinity and the combined stress, while *CATa* was highly significantly up-regulated under the combined stress, whereas the *APXa* was highly significantly up-regulated under the salinity stress. The transcript levels of these genes under different treatments are somewhat consistent with the activity of their corresponding enzymes activity (Figure 3A–C). Under the unstressed conditions, priming with 0.5 mM GABA up-regulated *SOD1*, *CATa* and *APXa* by 62.26, 17.00 and 19.17-fold, respectively as compared with the control condition. The transcription level of *SOD1* was up-regulated in the GABA-primed seeds under the osmotic stress, salinity and OS+S by 22.24, 24.82 and 32.33-fold, *CATa* by 70.04, 69.64, and 64.74-fold; and *APXa* by 32.13, 63.37 and 45.67-fold, respectively (Figure 3D–F). Similarly, priming seeds with 0.5 mM GABA resulted in up-regulation of the *PAL1*, *PPO* and *SKDH* genes under osmotic stress, salinity and OS+S stress relative to their controls (Figure 3J–L). The transcription level of *PAL1* and *SKDH* greatly increased under salinity and combined stress without significant differences between them. In contrast, *PPO* expression was greatly increased under the combined stress. In the absence of stress conditions, priming with 0.5 mM GABA resulted in up-regulation of the *PAL1*, *PPO* and *SKDH* genes by 3.06, 2.85 and 8.00-fold, respectively, as compared with the control condition. The relative expression level of *PAL1*, *PPO* and *SKDH* genes was up-regulated in the primed seedlings under the osmotic stress, by 50.07, 50.57 and 47.72-fold; salinity by 32.39, 38.15 and 32.81-fold, and OS+S by 28.38, 32.53 and 27.58-fold, respectively, as compared with their controls (Figure 3J–L). The transcription levels of *ibCAD1* (Figure 4D) and *Chi2* (Figure 4F) in the GABA-primed plants were highly significantly up-regulated when the seedlings were exposed to salinity stress as compared to osmotic stress and the combined stress, while the transcription level of *SbGST* was highly significantly up-regulated in the GABA-primed plants when exposed to osmotic stress as compared with salinity and combined stress (Figure 4E). Osmotic stress, salinity, and OS+S, priming with 0.5 mM GABA up-regulated *ibCAD1* by 36.55, 41.66 and 29.77-fold; *SbGST* by 64.19, 25.14, and 36.91-fold; and *Chi2* by 48.70, 64.02 and 60.46-fold, respectively, as compared with the controls (Figure 4D–F). The results reported that the *OsCIPK* genes in the GABA-primed seedlings were up-regulated under the osmotic, salinity and OS+S conditions as compared to the controls (Figure 5). Salinity stress resulted in significant increases in the expression level of the *OsCIPK01*, *OsCIPK03*, *OsCIPK08* and *OsCIPK15* genes as compared to osmotic stress and the combined stress (Figure 5A,C,E,H). Osmotic stress resulted in a greater increase in the expression levels of *OsCIPK02*, *OsCIPK07* and *OsCIPK09* genes as compared with salinity and the combined stress (Figure 5B,D,F), whereas the combined stress (OS+S) resulted in a significant up-regulation of *OsCIPK12* and *OsCIPK17* genes as compared with salinity and osmotic stress (Figure 5G,I).

### 2.10. Effects of GABA Priming on the Nuclear DNA Content and Ultramorphology of the Cell under Salinity, Osmotic Stress and OS+S 

**Figure 6:**
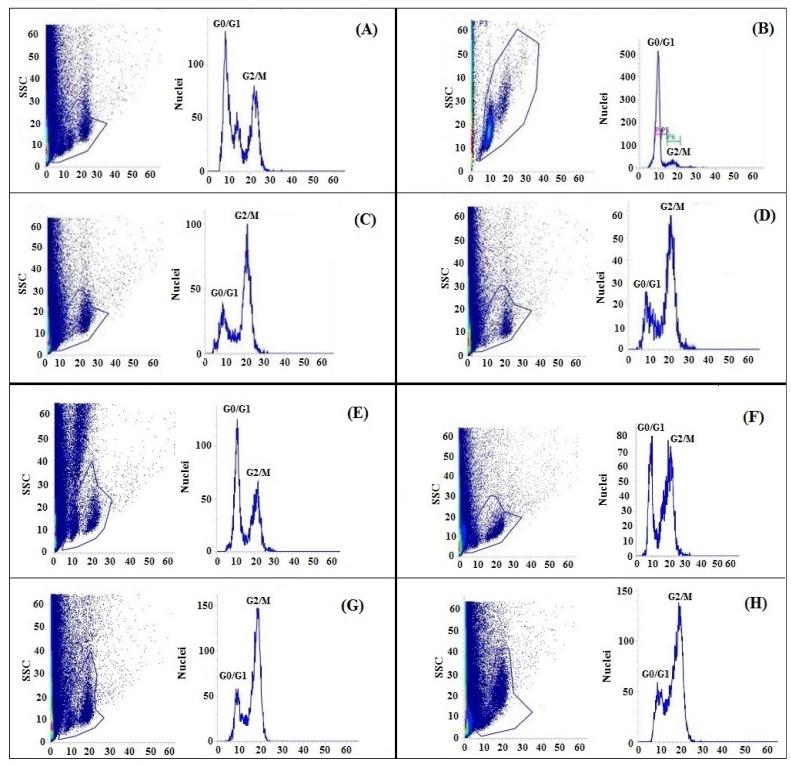
Flow cytometric analysis of GABA-unprimed rice (**A**–**D**) showing the nuclear DNA content of the root cell under control (**A**); osmotic stress (**B**); salinity (**C**) and their combined stress (**D**) and GABA-primed rice (**E**–**H**) under control (**E**); osmotic stress (**F**); salinity (**G**) and their combined stress (**H**).

**Figure 7:**
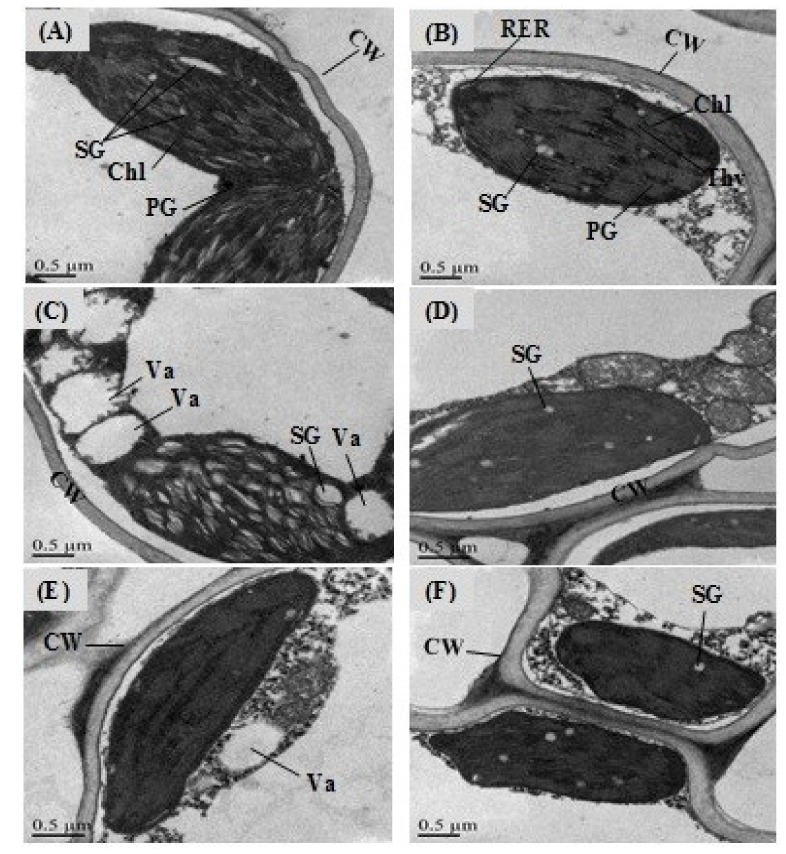
Transmission electron microscopic images of the leaf mesophyll of rice seedlings primed with 0.5 mM of GABA and grown under the control, salinity and the combination stress (OS+S) conditions. Control condition (**A**); primed with 0.5 mM GABA (**B**); salinity stress (**C**); salinity stress and primed with 0.5 mM GABA (**D**); combined stress (OS+S) (**E**); Combined stress (OS+S) and primed with 0.5 mM of GABA (**F**).

In order to investigate whether the osmotic stress, salinity and their combination could inhibit cell cycle progression, the nuclear DNA content was analyzed using the flow cytometry technique. The results showed that seedlings without GABA priming under osmotic stress and salinity and their combination underwent changes in cell cycle progression (Figure 6A–D). Under the osmotic stress condition (Figure 6B), the cells were blocked in at the G2/M phase, but the cells showed pronounced nuclear accumulation at the G0/G1 phase. However, with GABA priming and under the osmotic stress (Figure 6F), the nuclear accumulation was more obvious in both phases of the cell cycle. The salinity and the OS+S stresses induced a pronounced cell accumulation in G2/M and a sharp inhibition in the cell progression through the G0/G1 phase irrespective of the priming treatment (Figure 6C,D,G,H). The flow cytometry analysis indicated that the G0/G1 phases were more sensitive under the salinity alone or the combined stress as compared to the osmotic stress. These findings suggested that the osmotic stress, salinity and their combination result in a longer time for cells to progress through the cell cycle. However, each treatment affects cell cycle progression in a different way. The cell ultrastructure was affected due to the priming, salinity and the osmotic stress treatments (Figure 7). Under the control condition (Figure 7A) and priming treatment (Figure 7B), the transmission electron microcopy (TEM) analysis showed clear cell walls and developed chloroplasts (Chl) with uniform thylakoids (Thy). Under the salinity and the combined stress (OS+S), in the unprimed plants (Figure 7C,E), an unclear cell wall, a lot of vacuoles and raptured chloroplasts were observed. However, under the salinity and the combined stress (Figure 7D,F), the priming treatment somewhat improved the cell structure, which was represented by a clear cell wall, developed starch grain and the absence of the vacuoles. These results suggested that the priming with 0.5 mM GABA led to changes in the cell cycle progression and cell ultramorphology as a kind of cell signaling under salinity, osmotic stress and their combination.

## 3. Discussion

Enhancing plant tolerance to abiotic stresses by inducing a stress-responsive gene pathway in transgenic plants is a promising approach [28]. Combined stress is a new kind of abiotic stress in plants that requires integration defense or cross-talk response, and the plants should be tested for their tolerance to a combination of different stresses prior to cultivation under field conditions. The present study reported that priming with 0.5 mM GABA improved the physiological parameters under salinity and osmotic stress and their combination as compared with unprimed plants (Table 1). During seed germination and the stress condition, GABA could improve starch catabolism and mobilization of sugar and amino acids which are necessary for seedling growth [29]. GABA can also improve the antioxidant system for mitigation of oxidative damage, and increase Na^+^/K^+^ transportation for osmotic regulation under salt stress [29]. Furthermore, GABA could maintain the hormones and mineral nutrients and reduce lipid peroxidation under different environmental stresses [30]. During the early stage of seed imbibition, the up-regulation of some germination-related genes contributed to the metabolic process prior to seed germination which could improve the germination and seedling growth under the stress condition [31]. The reduction in rice growth from seed germination to the maturity stage under salinity stress may be due to the increase in osmotic pressure of the root medium and ion effects [32]. Several studies have reported that osmotic stress significantly decreased fresh and dry weights of shoots [33] and roots [34]. It has been found that exogenous GABA could significantly improve the shoot length, root length, and fresh weight of maize seedlings [35] and white clover [29] under abiotic stresses. In the present study, GABA induced starch catabolism, which is of primary importance for providing available carbohydrates for seed germination and growth under osmotic stress, salinity and the combined stress. These findings are consistent with previous studies, which found that environmental stresses such as osmotic stress, salt and heat stress decreased seed germination due to the inhibition of starch catabolism under these conditions [29,36,37]. It has been found that the metabolite mobilization of starch and soluble sugars is critical for the maintenance of cell turgor and energy sources when seeds are subjected to salinity and osmotic stress [36]. Additionally, GABA could increase starch catabolism and provide available carbohydrates for seed germination and growth of white clover under salt stress [29]. Moreover, starch catabolism could be accelerated by activating α- and β-amylase activities in GABA-pretreated seeds [29]. It is well-known that both organic and inorganic osmolytes such as sugars and starch are important osmotic regulators for plant adaption to environmental stresses [38,39,40]. These osmolytes were decreased in the present study under salinity, osmotic stress and OS+S, and were enhanced by priming with 0.5 mM GABA (Table 3). It has been found that the accumulation of osmolytes such as free starch, soluble sugars and protein regulated the osmotic pressure of the plants under abiotic stress including salinity and osmotic stress [41]. Furthermore, plants under stress conditions may accumulate small molecular weight proteins that could be used as a source of storage nitrogen and could be rapidly mobilized when required for the alleviation of stress [42]. These proteins could also have a role in osmotic adjustment [43]. Additionally, proline can also accumulate in plants to act as a solute for adaptation of plants in response to different environmental stresses [44]. 

The photosynthetic response to abiotic stress, especially salinity and osmotic stress, is highly complex. It involves the interaction of stress signaling with different plant cells which can promote plant growth and development. In the present study, rice plants grown under salinity, osmotic stress and OS+S treatments showed a marked reduction in Pn, gs and Tr (Table 2). The remarkable reduction in gs may be a consequence of stomatal closure caused by the higher osmotic pressure in guard cells under OS+S [45]. Another study observed a decrease of the chlorophyll content in rice under water stress, which may be related to the inhibition of the photosynthetic system of the plant under the water stress [46], or might be due to increases in ROS and lipid peroxidation levels leading to chlorophyll damage and a change in the leaf color from green to yellow [47]. In the present study, RWC was decreased by the salinity and osmotic stress and OS+S, and enhanced by GABA priming as compared with the control condition (Table 2). A decrease in the RWC under PEG-induced osmotic stress was also reported in rice leaves [46] and in tomato [48]; this might be due to the decreased water potential under salinity and osmotic stresses [49]. Similarly, the cellular accumulation of GABA could achieve a balance in the reduction of water potential that occurs during cellular dehydration under the stress condition [50]. 

Rice controls the transport of salts initially by selective uptake by root cells and ions entering into the root along with water through symplastic and apoplastic routes [51]. The ratio of Na^+^/K^+^ can be used as a physiological index for salt response in several crop plants such as tomato (*Solanum lycopersicum*) [52], chickpea (*Cicer arietinum*) [53], barley (*Hordeum vulgare*) [54] and white clover (*Trifolium repens*) [29]. Moreover, the concentration of Na^+^ is key for the salinity tolerance mechanism, interacting with K^+^ homeostasis, and especially given its involvement in numerous metabolic processes, maintaining a balanced cytosolic Na^+^/K^+^ ratio [55]. In the present study, priming with 0.5 mM GABA under salinity, osmotic stress and OS+S caused a significant reduction in the Na^+^ concentration in the leaf and root as compared with unprimed seeds (Figure 2). Under the salinity stress, plant cell loses the balance in the Na^+^/K^+^ ratio due to a continuously increasing Na^+^ ion concentration and decrease in K^+^ ion uptake [56]. In the current study, a higher concentration of Na^+^ was recorded in roots as compared to the leaf under the salinity and osmotic stress and their combination (Figure 2). The high accumulation of Na^+^ especially in the leaf resulted in a reduction in the photosynthesis due to stomatal and non-stomatal limitation [57]. Therefore, plants have to reduce the concentration of Na^+^ in the plant leaves by either minimizing the entry from the root symplast to reduce loading, maximizing Na^+^ retrieval from the xylem [58], or exporting Na^+^ from the leaf to the phloem [59]. As such, the down-regulation of genes encoding Na^+^ influx transporters (*OsCNGC1*) in rice root significantly contributed to the salinity tolerance, as it could avert toxic Na^+^ influx [60].

In the present study, antioxidant enzymes were measured to investigate the capability of priming with 0.5 mM GABA to induce such enzymes for mitigating the oxidative stress by scavenging ROS in the cell and thus increase the rice tolerance under the salinity, osmotic stress and their combination. In the current work, salt and osmotic stress and their combination (OS+S) significantly decreased the activity of CAT, SOD and APX (Figure 3), which was consistent with that of barely exposed to salinity stress [21]. However, the decrease in these enzymes was inhibited when the seeds were primed with 0.5 mM of GABA under the stress conditions (Figure 3). Similar findings were observed in different plant species primed with GABA such as rice seedlings [61], black pepper seedlings [62], perennial ryegrass [63] and white clover [29]. The accumulation of ROS and oxidative stress signal are the main common mechanisms for the plant tolerance could be observed under the salinity and osmotic stresses. The intensive accumulation of ROS in the different plant cells resulted in significant pigment loss, a reduction in the photosynthetic system efficiency and decreased protein assimilation [64]. However, induction of the antioxidant defense system could protect plants from oxidative damage induced by ROS accumulation under stress conditions [65]. In the present study, salinity and osmotic stress and OS+S improved the ROS, i.e., H_2_O_2_, O_2_^−^ and OH^−^ accumulation in the leaf tissue as compared with the control condition (Table 2). The present study reported that GABA-activated antioxidant enzymes could play vital roles in scavenging free radicals induced by H_2_O_2_ and O_2_^−^ and reduced lipid peroxidation by up-regulating the genes (*APXa*, *CATa* and *SOD1*) involved in antioxidant enzymes during rice seed germination and seedling growth under salinity and osmotic stress and their combination (Figure 3). 

The accumulation of phenolic compounds under environmental stresses can protect plants from damage caused by ROS-induced oxidative stress [66] by scavenging free radicals, breaking radical chain reactions, and decomposing peroxides [67]. In the present study, the enzymes involved in the phenolic metabolism such as PAL, PPO, SKDH and CAD were improved by priming with 0.5 mM GABA under the osmotic, salinity and OS+S stresses (Figure 3G–I and Figure 4A). This phenolic metabolism may provide an effective defense tool for plant tolerance under environmental stresses [68]. Recently, SKDH activity was increased in plants exposed to salinity stress for 3 days [69]. Moreover, the activity and the expression level of the *PAL* protein were increased in barley exposed to 200 µM of Al for 24 h [70]. The increased PAL and PPO levels might help plants to cope with oxidative stress by scavenging ROS [45,71]. In the present study, the accumulation of PPO in the leaf under salinity and osmotic stresses (Figure 3H), might be due to the induction of *PPO* genes in the leaf tissues under osmotic stress [72]. However, the PPO and CAD activities were not affected in *Matricaria chamomilla* plants exposed to salinity stress, but a higher activity of SKDH was observed in the root [68]. In the present study, the induction of the defense genes involved in secondary metabolites such as *PAL1*, *PPO*, *SKDH* and *IbCAD1* in the primed plants may increase the tolerance of plants to salinity, osmotic stress and OS+S (Figure 3J–L and Figure 4B), which is consistent with the finding of Ahammed et al. [71], who also found that plant growth regulators help in alleviating the oxidative stress in plant leaves through the induction of phenolic metabolism defense under the stress condition. The mechanism by which GABA treatment increases the tolerance to salt stress might be due to the ability of GABA to induce endogenous GABA, proline and the total phenolic content, thus enhancing the antioxidant capacity [67]. The present study revealed that the GST and chitinase activities and their transcript levels were increased by priming with 0.5 mM of GABA under salinity, osmotic stresses and their combination (Figure 4). Similarly, a previous study reported that the *Chi2* gene was up-regulated in pepper leaves under salinity and osmotic stresses which could protect plant tissues against osmotic stress via an ABA-independent signal transduction pathway [73]. Another study reported that chitinase is involved in heavy metal stress tolerance and chilling tolerance [74]. Moreover, GST activity and expression level increased in salinity and osmotic -stressed barley plants [45].

In the present study, up-regulation of *OsCIPK* genes was observed in the GABA-primed seedlings under salinity and osmotic stress and OS+S as compared to their controls (Figure 5). The *OsCIPK* genes induced by different stresses may provide new signaling pathway to reveal the molecular mechanism of rice response to different stresses alone or in combination considering the nature of *CIPKs* as putative signaling components [75]. The signaling pathway of *OsCIPK* genes may be involved in the substantial common regulatory systems or cross talks triggered by different stresses [75]. Our findings indicated that expression patterns of *OsCIPK* genes were induced under the salinity, osmotic stress and OS+S, which is consistent with cross talk between salinity and osmotic stress as previously reported by Seki et al. [76]. The interaction between co-activated pathways is likely to be mediated at different levels under the combined stresses [13]. This pathway could include the interaction between different transcription factors and mitogen-activated protein kinase (MAPK) cascades [77], different stress hormones such as ethylene, jasmonic acid and abscisic acid [78], between calcium and/or ROS signaling [79] and between different receptors and signaling complexes [80]. 

The present study revealed that nuclear accumulation was inhibited under the stress condition especially under salinity and the combined stress in the G0/G1 as compared with the control or osmotic stress (Figure 6). A recent study showed that root growth was inhibited under the abiotic stress conditions, and the cell division and cell cycle regulation might be involved in this inhibition [80], or might be associated with the reduction of cell production [81]. The reduced cell production under the salinity and osmotic stress and their combination might be due to a smaller number of dividing cells such as a meristem size reduction, and the temporary inhibition of mitotic activity that allows the adaptation to the stress condition is most likely mediated by post-translational control of cyclin-dependent kinase activity (CDK) [80]. 

## 4. Materials and Methods

### 4.1. Plant Materials and Growth Conditions

Rice (*Oryza sativa* cv. Qian You No. 0508) seeds were purchased from the Seed Production Unit, Jiangsu Academy of Agricultural Sciences, China. Before priming, seeds were sterilized with 0.5% NaClO solution for 15 min and washed several times to remove the traces of the disinfectant. The seeds were then primed with GABA at the optimized concentration (0 and 0.5 mM) at 15 °C in darkness for 24 h. The time and concentration of the priming agent were initially selected based on a preliminary study. In the preliminary experiment, several concentrations of GABA, i.e., 0, 0.1, 0.2, 0.3, 0.4, 0.5, 0.6 and 0.7 mM were used for seed priming. GABA at 0.5 mM significantly improved rice germination and seedling growth as compared to other concentrations. The seed were primed for 24 h as this time was sufficient enough for rice seeds to trigger the activation of various metabolic processes such as the synthesis of hydrolytic enzymes which resulted in hydrolysis of reserve food into a simple available form for embryo uptake as stated recently in our previous study [82,83,84,85]. The primed seeds were dried at room temperature to maintain their original moisture content [82]. Thereafter, the primed and unprimed seeds were germinated for two weeks in a plastic germination box containing two layers of germination paper moistened with water. Fifty seeds and three replications for each treatment were used. Then, seeds were incubated in a germination chamber at 25 °C with 80 % relative humidity under alternating cycles of 16 h illumination and 8 h darkness for 14 days. 

The salinity and osmotic stress and their combination were applied to 7-day-old rice seedlings, in which the salinity stress (150 mM NaCl) and the osmotic stress (30 g/L PEG, 6000) and their combination (150 mM NaCl+30 g/L PEG) were supplied to plants for one week. The seedlings without GABA priming and stress treatments were used as the control (Ck). After fourteen days, the germination percentage, germination energy, root and shoot length, seedling fresh and dry weight and seedling vigor index were measured according to the methods of Hu et al. [86]. 

### 4.2. Determination of Photosynthesis and Leaf water Relation

The physiological parameters such as Pn, Tr, Gs and Ci were measured according to our previous study [87]. The chlorophyll content was measured spectrophotometrically according the method of Sheteiwy et al. [88]. The values of Ψw, WUE and RWC were measured according to our previous study [87]. The Ψs was measured according to the method of Ahmed et al. [8].

### 4.3. Biochemical Analysis

For further investigation regarding the potential role of GABA priming to alleviate the oxidative stress induced by salinity and osmotic stress, the antioxidant enzymes, i.e., SOD, CAT and APX were measured according the method of Sheteiwy et al. [88]. GR and total soluble sugars were measured according the method of Sheteiwy et al. [89]. The starch content and total soluble proteins were measured according to the method of Sheteiwy et al. [90]. 

### 4.4. Analysis of MDA, Proline and ROS Contents 

The MDA content of the leaf was measured according to the method of Zhou and Leul [91]. Proline concentration was determined by a spectrophotometer according to the method of Li [92]. Briefly, 100 mg of leaf was homogenized with 5 mL of 3% sulfosalicylic acid and centrifuged at 5000× *g* for 10 min. The supernatant was treated with acid-ninhydrin and acetic acid, after which the supernatant was boiled for 1 h at 100 °C. Absorbance was determined at 520 nm and the proline content was expressed as μg gFW^−1^. For determination of the H_2_O_2_ content, 0.5 g of leaf was homogenized with 5.0 mL of 0.1% trichloroacetic acid (TCA) using an ice bath, and then the homogenate was centrifuged for 15 min at 12,000× *g* [93]. The H_2_O_2_ content in the supernatant was read using a spectrophotometer at 390 nm. The content of O_2_^−^ was measured according to Jiang and Zhang’s method [94]. The content of OH^−^ in the leaf was determined according to our previous study [89]. 

### 4.5. Determination of GABA Content

The GABA content in the leaf tissues was measured using GABAase commercial enzyme preparation (Sigma chemical Co., St. Louis, MI, USA) as previously described by Ma et al. [21]. 

### 4.6. Assay of Phenolic Metabolism-related Enzymes

The activity of PAL was determined according to the method of Zheng et al. [95] with slight modification. Fresh leaves (2g) were homogenized with 2.5 mL of solution containing 100 mM K_3_PO_4_ buffer, 2 mM EDTA, 1% (*m*/*v*) PVP, and 1 mM phenyl-methylsulfonyl fluoride (PMSF). Then, the homogenates were centrifuged at 12,000× *g* for 15 min at 4 °C, and the supernatant fractions were used for enzyme analysis. The absorbance was spectrophotometrically measured at 290 nm. The activities of PPO and SKDH were measured according to the method of Sheteiwy et al. [82]. The activity of CAD was measured according to the method of Wyrambik and Grisebach [96].

### 4.7. Measurements of Detoxification-related Enzymes

In order to determine the activity of GST, 0.3 g of leaf tissue was homogenized with 2 mL phosphate buffer solution (pH 6.5) + 1 mM EDTA. The suspension was centrifuged at 4000× *g* for 10 min. The GST activity was measured spectrophotometry at 412 nm following the method of Chun-hua and Ying [97]. The chitinase activity was measured according to the method of Chun-hua and Ying [97].

### 4.8. Measurement of Na^+^ and K^+^ Ions in the Leaf and Root Tissues 

The concentrations of Na^+^ and K^+^ in leaf and root were measured according to the methods of Zhao et al. [98]. Briefly, the samples were washed with distilled water before the determination to remove any traces of the Na^+^ from the leaf and root tissues, which were then dried at 50 °C for 4 d. Thereafter, the dried leaf and root tissues were ground into a fine powder in liquid nitrogen. The powder was then digested in 5 mL nitric acid overnight. Thereafter, the digested solution was diluted to 25 mL with double-distilled water. The concentration of Na^+^ and K^+^ in the acid-digested tissues was measured using a flame photometer according to the method of Zhao et al. [98].

### 4.9. Analysis of Gene Expression 

In order to further study the mechanism by which GABA can alleviate the effects of both salinity and osmotic stresses alone or in combination on rice germination and seedling growth, the antioxidant enzymes, detoxification-related enzymes, phenolic metabolism-related enzymes and *OsCIPK* responses genes were investigated at the molecular levels. For this purpose, frozen leaf tissues (100 mg each) were ground thoroughly in liquid nitrogen using a pestle and mortar. Thereafter, the total RNA was isolated from the leaf and the concentration of the RNA was determined by a NanoDrop 2000/2000c (Thermo Scientific, Wilmington, Delaware, USA). The RNA purity was also checked by the spectrophotometer using the 260/280 nm ratio before quantitative real-time PCR. The primers of the *OsCIPK* genes presented in Supplementary Table S1 are the same as those used previously by Xiang et al. [75]. Quantitative real-time RT-PCR was performed using SYBR premix EX Taq (Takara, Japan). The PCR program used in this study is the same as that used recently by Sheteiwy et al. [88].

### 4.10. Ultra-structure and Flow Cytometry Analysis 

The ultramorphology of the leaf was investigated according to our previous study [82]. The H_2_O_2_ was detected according to the method of Sheteiwy et al. [82]. Briefly, the roots were stained with 5 μM dichlorodihydrofluorescein diacetate for 15 min, and then washed with excess 20 mM sodium phosphate buffer (pH 6.1) to stop the reaction. The changes in ΔΨm were analyzed using a tetramethylrhodamine methyl ester assay kit (Immunochemistry Technologies, Bloomington, IN, USA) and imaged using a laser confocal scan microscope (Zeiss LSM 780, Zeiss, Germany). Then, nuclear isolation was performed according to the method of Hu et al. [99]. The root samples were cut into small pieces and then fixed with nucleus isolation buffer [10 mM MgSO_4_, 50 mM KCl, 5 mM Hepes, 1 mg/mL dithiothreitol (Sigma, St. Louis, MI, USA) and 0.2% Triton X-100] and filtered through a 33 mm nylon mesh. The nuclei were fixed in 4% paraformaldehyde for 30 min and were then precipitated (200 g, 10 min, 4u C) and re-suspended in the isolation buffer.

### 4.11. Experimental Design and Statistical Analysis

The treatments were applied using a Completely Randomized Block Design (CRBD) with a factorial arrangement. All the obtained values are the means of three replicates ± standard deviation (SD). The data were analyzed using two-way analysis of variance (ANOVA) by SPSS v16.0 (SPSS, Inc., Chicago, IL, USA), and means were separated using Duncan’s multiple range tests (α = 0.05).

## 5. Conclusions

Priming with 0.5 mM of GABA could be an effective technique to alleviate salinity and osmotic stresses and OS+S causing inhibition of rice seed vigor. Under the stress conditions, GABA induced a balance in Na^+^/K^+^ accumulation and transport from the root to the leaf which could be attributed to the osmotic adjustment through the mobilization of organic osmolytes such as proline, sugars, and starch during seed germination. The fluorescence staining revealed that H_2_O_2_ formation was increased under the stress condition and decreased by the GABA priming treatment. These findings indicated that GABA could also act as a signal molecule under salinity, osmotic stress and their combination by increasing antioxidant enzymes, phenolic metabolism-related enzymes and detoxification-related enzyme activities and their transcript levels. The significantly improved starch and sugar contents and *CIPK* gene expression in rice seedlings by GABA treatment under the stress conditions may be the main mechanism of rice tolerance to salinity, osmotic stress and their combination. The present study elucidated the possible cross talk between the salinity and osmotic stresses when the plant are exposed to both at the same time, and thus to develop transgenic crops with enhanced tolerance to field conditions, further studies need to expand their area to include stress combinations. Current findings provide new evidence for better understanding of GABA-regulated osmotic and salinity combined stress tolerance during seed germination and development. The results showed that the different abiotic stresses induced changes in cell cycle progression resulting in inhibition in rice root cell development. Priming with 0.5 mM GABA has the potential to improve cell ultra-morphology under the stress condition. 

## Figures and Tables

**Table 1:** Effects of GABA treatment on the germination percentage (GP) germination energy (GE), root length (RL, cm), shoot length (SL, cm), seedlings fresh weight (SFW, g), seedling dry weight (SDW, g) and seedling vigor index (SVI) of rice seedlings exposed to salinity, osmotic stress and their combined stress.

Treatments	GP	GE	RL	SL	SFW	SDW	SVI
Ck	88.66 ± 0.57a	80.66 ± 3.78ab	12.40 ± 0.17b	7.63 ± 5.41a	0.160 ± 0.01b	0.062 ± 0.002b	1775 ± 459ab
Osmotic (OS)	84.66 ± 0.41b	79.33 ± 4.72bc	9.53 ± 0.25d	7.50 ± 0.30a	0.126 ± 0.07b	0.057 ± 0.002bc	1443 ± 106bc
Salinity (S)	76.66 ± 0.35de	57.66 ± 0.57e	7.60 ± 0.45f	6.56 ± 0.37a	0.096 ± 0.02b	0.049 ± 0.003de	1085 ± 53cd
OS+S	73.66 ± 0.35e	52.00 ± 2.00f	6.56 ± 0.37g	6.50 ± 0.50a	0.061 ± 0.04c	0.035 ± 0.001f	962 ± 65d
Priming (P)	89.00 ± 1.00a	85.33 ± 0.57a	14.30 ± 0.62a	7.63 ± 5.40a	0.192 ± 0.02a	0.086 ± 0.002a	1948 ± 518a
OS+P	88.33 ± 1.15a	81.66 ± 2.08ab	10.78 ± 0.36c	8.33 ± 0.11a	0.131 ± 0.03b	0.057 ± 0.006bc	1688 ± 14ab
S+P	80.33 ± 0.57c	75.66 ± 0.57c	8.50 ± 0.40e	7.70 ± 0.26a	0.114 ± 0.05b	0.053 ± 0.003cd	1301 ± 20b–d
OS+S+P	79.00 ± 0.37cd	69.66 ± 3.51d	9.40 ± 0.36d	9.00 ± 0.45a	0.092 ± 0.01b	0.044 ± 0.001e	1453 ± 36bc

Values are means ± SD (*n* = 3) and the same letters within a column indicate no significant difference at a 95% probability level (*p* < 0.05).

**Table 2:** Effects of GABA treatment on the net photosynthetic rate (Pn), transpiration rate (Tr), stomatal conductance (Gs), intracellular CO_2_ concentration (Ci); chlorophyll content (SPAD), water potential (WP, Ψw), osmotic potential (OP, Ψs), relative water content (RWC) and water use efficiency (WUE) of rice seedlings exposed to salinity, osmotic stress and their combined stress.

Treatments	Pn	Tr	Gs	Ci	SPAD	WP (Ψw)	OP (Ψs)	RWC	WUE
Ck	6.67 ± 0.15b	3.60 ± 0.20b	0.613 ± 0.47b	417.67 ± 35.30a	11.70 ± 0.55b	−2.36 ± 0.15ab	−0.14 ± 0.01b	33.04 ± 2.15b	1.59 ± 0.19f
Osmotic (OS)	3.46 ± 0.11d	1.70 ± 0.10e	0.453 ± 0.06c	225.00 ± 5.00b	6.33 ± 0.15e	−6.68 ± 0.17e	−0.17 ± 0.01b	11.56 ± 1.48f	3.38 ± 0.23d
Salinity (S)	2.84 ± 0.09e	1.23 ± 0.05f	0.260 ± 0.03e	115.00 ± 5.00d	7.98 ± 0.45d	−9.43 ± 0.90f	−0.38 ± 0.04d	16.81 ± 1.37e	2.36 ± 0.07e
OS+S	1.86 ± 0.13f	0.88 ± 0.02g	0.173 ± 0.02f	136.67 ± 7.63cd	8.20 ± 0.36d	−13.40 ± 0.95g	−0.26 ± 0.02c	9.68 ± 0.50f	2.17 ± 0.12e
Priming (P)	11.10 ± 0.52a	5.46 ± 0.30aa	1.51 ± 0.05a	410.00 ± 65.57a	16.37 ± 0.12a	−1.60 ± 0.16a	−0.08 ± 0.01a	63.61 ± 2.60a	6.83 ± 0.10a
OS+P	5.60 ± 0.19c	3.07 ± 0.06c	0.543 ± 0.05b	248.33 ± 10.40b	8.70 ± 0.85d	−3.69 ± 0.13c	−0.14 ± 0.01b	25.86 ± 2.53c	5.11 ± 0.41b
S+P	3.73 ± 0.12d	2.73 ± 0.07d	0.426 ± 0.02c	175.67 ± 4.04c	9.60 ± 0.17c	−2.93 ± 0.06bc	−0.24 ± 0.01c	30.51 ± 1.17b	4.16 ± 0.12c
OS+S+P	2.77 ± 0.19e	1.78 ± 0.17e	0.343 ± 0.03d	154.33 ± 5.13cd	9.80 ± 0.43c	−4.91 ± 0.61d	−0.26 ± 0.01c	22.78 ± 0.67d	3.52 ± 0.07d

Values are means ± SD (*n* = 3) and the same letters within a column indicate no significant difference at a 95% probability level (*p* < 0.05).

**Table 3:** Effects of GABA treatment on the GABA content (mg/g DW), sugars (µg/g FW), protein (mg/g FW), starch (%); proline (µg/g FW); hydrogen peroxide (H_2_O_2,_ nmol g^−1^ FW), superoxide radical (O_2_^−^, nmol min^−1^g^−1^ FW), hydroxyl ion (OH^−^, nmol g^−1^ FW); malonaldehyde (MDA, nmol mg^−1^ protein) and glutathione reductase (GR, µmol min^−1^ mg^−1^ protein) of rice seedlings exposed to salinity, osmotic stress and their combined stress.

Treatments	GABA Con.	Sugars	Protein	Starch	Proline	H_2_O_2_	O_2_^−^	OH^−^	MDA	GR
Ck	3.53 ± 0.10g	12.50 ± 0.17a	240.0 ± 8.6b	66.37 ± 0.89b	30.35 ± 2.07c	1.64 ± 0.06de	2.59 ± 0.15c	0.103 ± 0.05d	81.75 ± 4.8e	6.72 ± 0.54f
Osmotic (OS)	5.71 ± 0.10d	6.30 ± 0.25d	108.35 ± 6.2e	33.25 ± 2.65de	48.55 ± 1.89b	3.52 ± 0.06b	2.68 ± 0.18c	0.153 ± 0.01b	105.60 ± 1.5cd	10.53 ± 0.07e
Salinity (S)	4.76 ± 0.24e	4.96 ± 0.20e	102.81 ± 6.3e	27.13 ± 1.47e	73.19 ± 2.51a	4.34 ± 0.18a	3.95 ± 0.50a	0.210 ± 0.04a	159.90 ± 3.5a	14.44 ± 0.42d
OS+S	4.52 ± 0.24e	4.17 ± 0.30f	85.17 ± 5.84f	28.84 ± 0.41e	76.44 ± 1.7a	4.15 ± 0.53a	3.49 ± 0.05b	0.156 ± 0.05b	125.46 ± 2.0b	17.31 ± 0.42c
Priming (P)	4.07 ± 0.06f	12.24 ± 0.30a	361.01 ± 12.9a	81.07 ± 7.62a	30.40 ± 1.15c	1.47 ± 0.21e	2.69 ± 0.34c	0.099 ± 0.05d	83.24 ± 2.65e	6.10 ± 0.18f
OS+P	10.45 ± 0.05a	9.90 ± 0.52b	160.0 ± 5.60d	63.90 ± 3.72b	34.56 ± 1.04c	1.98 ± 0.01d	2.67 ± 0.27c	0.126 ± 0.05b-d	83.38 ± 10.4e	18.61 ± 0.82bc
S+P	8.42 ± 0.39b	9.00 ± 0.46c	215.37 ± 5.0c	48.82 ± 3.26c	41.68 ± 1.70bc	2.69 ± 0.24c	2.73 ± 0.07c	0.136 ± 0.05bc	112.02 ± 2.7c	20.00 ± 0.21b
OS+S+P	6.60 ± 0.16c	10.19 ± 0.54b	168.86 ± 16.3d	39.47 ± 4.91d	49.18 ± 6.30b	2.95 ± 0.07c	2.55 ± 0.09c	0.116 ± 0.01cd	102.67 ± 3.8d	27.68 ± 2.00a

Values are means ± SD (*n* = 3) and the same letters within a column indicate no significant difference at a 95% probability level (*p* < 0.05).

## Supplementary Materials

Supplementary materials can be found at https://www.mdpi.com/1422-0067/20/22/5709/s1.

## References

[1] Blumwald E. (2000). Sodium transport and salt tolerance in plants. Curr. Opin. Cell Biol..

[2] Munns R., Tester M. (2008). Mechanisms of salinity tolerance. Ann. Rev. Plant Biol..

[3] Rajendran K., Tester M., Roy S.J. (2009). Quantifying the three main components of salinity tolerance in cereals. Plant Cell Environ..

[4] Akbar M., IRRI (1986). Breeding for salinity tolerance in rice. Salt-affected Soils of Pakistan, India and Thailand.

[5] Zhu G.Y., Kinet M., Lutts S. (2001). Characterization of rice (*Oryza sativa* L.) F3 populations selected for salt resistance. I. Physiological behaviour during vegetative growth. Euphytica.

[6] Castillo E.G., Tuong T.P., Ismail A.M., Inubushi K. (2007). Response to salinity in rice: Comparative effects of osmotic and ionic stresses. Plant Prod. Sci..

[7] Kaiser W.M. (2009). Effects of water deficit on photosynthetic capacity. Plant Physiol..

[8] Ahmed I.M., Dai H., Zheng W., Cao F., Zhang G., Sun D., Wu F. (2013). Genotypic differences in physiological characteristics in the tolerance to drought and salinity combined stress between Tibetan wild and cultivated barley. Plant Physiol. Biochem..

[9] Wang R., Chen S., Zhou X., Shen X., Deng L., Zhu H., Shao J., Shi Y., Dai S., Fritz E.P. (2008). Ionic homeostasis and reactive oxygen species control in leaves and xylem sap of two poplars subjected to NaCl stress. Tree Physiol..

[10] Singh N.P., Pal P.K., Vaishali S.K. (2014). Morpho-physiological characterization of Indian wheat genotypes and their evaluation under drought condition. Afr. J. Biotechnol..

[11] Turkan I., Bor M., Ozdemir F., Koca H. (2005). Differential responses of lipid peroxidation and antioxidants in the leaves of drought-tolerant P. acutifolius Gray and drought-sensitive P. vulgaris L. subjected to polyethylene glycol mediated water stress. Plant Sci..

[12] Wu Y., Yang C. (2016). Physiological responses and expression profile of NADPH oxidase in rice (*Oryza sativa*) seedlings under different levels of submergence. Rice.

[13] Mittler R. (2006). Abiotic stress, the field environment and stress combination. Trends Plant Sci..

[14] Rizhsky L., Liang H., Shuman J., Shulaev V., Davletov S., Mittler R. (2004). When defense pathways collide: The response of Arabidopsis to a combination of drought and heat stress. Plant Physiol..

[15] Fowler S., Thomashow M.F. (2002). Arabidopsis Transcriptome profiling indicates that multiple regulatory pathways are activated during cold acclimation in addition to the CBF cold response pathway. Plant Cell.

[16] Kinnersley A.M., Turano F.J. (2000). Gamma aminobutyric acid (GABA) and plant responses to stress. Crit. Rev. Plant Sci..

[17] Mekonnen D.W., Flüggea U., Ludewig F. (2016). Gamma-aminobutyric acid depletion affects stomata closure and drought tolerance of *Arabidopsis thaliana*. Plant Sci..

[18] Jordan B.R., Givan C.V. (1979). Effects of light and inhibitors on glutamate metabolism in leaf discs of *Vicia faba* L.. Plant Physiol..

[19] Bown A.W., MacGregor K.B., Shelp B.J. (2006). Gamma-aminobutyrate: Defense against invertebrate pests. Trends Plant Sci..

[20] Bouché N., Fromm H. (2004). GABA in plants: Just a metabolite. Trends Plant Sci..

[21] Ma Y., Wang P., Wang M., Sun M., Gu Z., Yang R. (2019). GABA mediates phenolic compounds accumulation and the antioxidant system enhancement in germinated hulless barley under NaCl stress. Food Chem..

[22] Renault H., Roussel V., Amrani A.E., Arzel M., Renault D., Bouchereau A. (2010). The Arabidopsis pop2-1 mutant reveals the involvement of GABA transaminase in salt stress tolerance. BMC Plant Biol..

[23] Barbosa J.M., Singh N.K., Cherry J.H., Locy R.D. (2010). Nitrate uptake and utilization is modulated by exogenous gamma-aminobutyric acid in Arabidopsis thaliana seedlings. Plant Physiol. Biochem..

[24] Beuve N., Rispail N., Laine P., Cliquet J.B., Ourry A., Le Deuneff E. (2004). Putative role of gamma-aminobutyric acid (GABA) as a long-distance signal in up-regulation of nitrate uptake in *Brassica napus* L.. Plant Cell Environ..

[25] Palanivelu R., Brass L., Edlund A.F., Preuss D. (2003). Pollen tube growth and guidance is regulated by POP2, an Arabidopsis gene that controls GABA levels. Cell.

[26] Deleu C., Faes P., Niogret M.F., Bouchereau A. (2013). Effects of the inhibitor of the g-aminobutyrate-transaminase, vinyl-gaminobutyrate, on development and nitrogen metabolism in Brassica napus seedlings. Plant Physiol. Biochem..

[27] Niu L., Dong B., Song Z., Meng D., Fu Y. (2018). Genome-wide identification and characterization of CIPK family and analysis responses to various stresses in apple (*Malus domestica*). Int. J. Mol. Sci..

[28] Kasuga M., Liu Q., Miura S., Yamaguchi-shinozaki K., Shinozaki K. (1999). Improving plant drought, salt, and freezing tolerance by gene transfer of a single stress-inducible transcription factor. Nat. Biotechnol..

[29] Cheng B., Li Z., Liang L., Cao Y., Zeng W., Zhang X., Ma X., Huang L., Nie G., Liu W. (2018). The γ-Aminobutyric Acid (GABA) alleviates salt stress damage during seeds germination of white clover associated with Na^+^/K^+^ transportation, dehydrins accumulation, and stress-related genes expression in white clover. In. J. Mol. Sci..

[30] Alqarawi A.A., Hashem A., AbdAllah E.F., Al-Huqail A.A., Alshahrani T.S., Alshalawi S.A.R., Egamberdieva D. (2016). Protective role of gamma amminobutyric acid on *Cassia italica* Mill under salt stress. Legume Res..

[31] He F., Shen H., Lin C., Fu H., Sheteiwy M.S., Guan Y., Huang Y., Hu J. (2017). Transcriptome analysis of chilling-imbibed embryo revealed membrane recovery related genes in maize. Front. Plant Sci..

[32] Grattan S., Zeng L., Shannon M., Robert S.R. (2002). Rice is more sensitive to salinity than previously thought. Calif. Agric..

[33] Centritto M., Lauteri M., Monteverdi M.C., Serraj R. (2009). Leaf gas exchange, carbon isotope discrimination, and grain yield in contrasting rice genotypes subjected to water deficits during the reproductive stage. J. Exp. Bot..

[34] Ji K.X., Wang Y.Y., Sun W.N., Lou Q.J., Mei H.W., Shen S.H., Chen H. (2012). Drought-responsive mechanisms in rice genotypes with contrasting drought tolerance during reproductive stage. J. Plant Physiol..

[35] Wang Y., Gu W., Yao M., Xie T., Li L., Jing L., Shi W. (2017). γ-Aminobutyric acid imparts partial protection from salt stress injury to maize seedlings by improving photosynthesis and upregulating osmoprotectants and antioxidants. Sci. Rep..

[36] Li Z., Peng Y., Zhang X.Q., Ma X., Hang L.K., Yan Y.H. (2014). Exogenous spermidine improves seed germination of white clover under water stress via involvement in starch metabolism, antioxidant defenses and relevant gene expression. Molecules.

[37] Fu Y., Gu Q., Dong Q., Zhang Z., Lin C., Hu W., Pan R., Guan Y., Hu J. (2019). Spermidine enhances heat tolerance of rice seeds by modulating endogenous starch and polyamine metabolism. Molecules.

[38] Ghoulam C., Foursy A., Fares K. (2002). Effects of salt stress on growth, inorganic ions and proline accumulation in relation to osmotic adjustment in five sugar beet cultivars. Environ. Exp. Bot..

[39] Hamoud Y.A., Wang Z., Guo X., Shaghaleh H., Sheteiwy M., Chen S., Qiu R., Elbashier M. (2019). Effect of Irrigation regimes and soil texture on the potassium utilization efficiency of rice. Agronomy.

[40] Hamoud Y.A., Shaghaleh H., Sheteiwy M., Guo X., Elshaikh N.A., Khan N., Oumarou A., Rahim S.F. (2019). Impact of alternative wetting and soil drying and soil clay content on the morphological and physiological traits of rice roots and their relationships to yield and nutrient use-efficiency. Agric. Water Manag..

[41] Trovato M., Mattioli R., Costantino P. (2008). Multiple roles of proline in plant stress tolerance and development. Rend. Lincei.

[42] Singh N.K., Bracker C.A., Hasegawa P.M., Handa A.K., Buckel S., Hermodson M.A. (1987). Characterization of osmotin: A thaumatin-like protein associated with osmotic adaptation in plant cells. Plant Physiol..

[43] Ashraf M., Harris P. (2004). Potential biochemical indicators of salinity tolerance in plants. Plant Sci..

[44] Wang C., Fan L., Gao H., Wu X., Li J., Lv G., Gong B. (2014). Polyamine biosynthesis and degradation are modulated by exogenous gamma-aminobutyric acid in root-zone hypoxia-stressed melon roots. Plant Physiol. Biochem..

[45] Ahmed I.M., Nadira U.A., Bibi N., Cao F., He X., Zhang G., Wu F. (2015). Secondary metabolism and antioxidants are involved in the tolerance to drought and salinity, separately and combined, in Tibetan wild barley. Environ. Exp. Bot..

[46] Hsu S.Y., Kao C.H. (2003). Differential effect of sorbitol and polyethylene glycol on antioxidant enzymes in rice leaves. Plant Growth Regul..

[47] Shivakrishna M.P., Reddy K.A., Rao D.M. (2018). Effect of PEG-6000 imposed drought stress on RNA content, relative water content (RWC), and chlorophyll content in peanut leaves and roots. Saudi J. Biol. Sci..

[48] Zgallaï H., Steppe K., Lemeur R. (2005). Photosynthetic, physiological and biochemical responses of tomato plants to polyethylene glycol-induced water deficit. J. Integr. Plant Biol..

[49] Seif S.N., Tafazzoli E., Talaii A., Aboutalebi A., Abdosi V. (2014). Evaluation of two grape cultivars (*Vitis vinifera* L.) against salinity stress and surveying the effect of methyl jasmonate and epibrassinolide on alleviation the salinity stress. In. J. Biosci..

[50] Heber U., Tyankova L., Santarius K.A. (1971). Stabilization and inactivation of biological membranes during freezing in the presence of amino acids. BBA Biomembr..

[51] Das I., Krzyzosiak A., Schneider K., Wrabetz L., Antonio M., Barry N., igurdardottir A., Bertolotti A. (2015). Preventing proteostasis diseases by selective inhibition of a phosphatase regulatory subunit. Res. Rep..

[52] Juan M., Rivero R.M., Romero L., Ruiz J.M. (2005). Evaluation of some nutritional and biochemical indicators in selecting salt-resistant tomato cultivars. Environ. Exp. Bot..

[53] Tejera N.A., Soussi M., Lluch C. (2006). Physiological and nutritional indicators of tolerance to salinity in chickpea plants growing under symbiotic conditions. Environ. Exp. Bot..

[54] Turkyilmaz B., Aktas Y., Guven A. (2011). Salinity induced differences in growth and nutrient accumulation in five barley cultivars. Turk. J. Field Crops.

[55] Assaha D.V.M., Uedam A., Saneoka H., Al-Yahyai R., Yaish M.W. (2017). The role of Na^+^ and K^+^ transporters in salt stress adaptation in glycophytes. Front. Physiol..

[56] Qui L., Wu D.Z., Ali S., Cai S., Dai F., Jin X., Wu F.B., Zhang G.P. (2011). Evaluation of salinity tolerance and analysis of allelic function of *HvHKT2* in Tibetan wild barley. Theor. Appl. Genet..

[57] Yeo A.R. (1998). Molecular biology of salt tolerance in the context of whole-plant physiology. J. Exp. Bot..

[58] Davenport R.J., Munoz-Mayor A., Jha D., Essah P.A., Rus A., Tester M. (2007). The Na^+^ transporter AtHKT1; 1 controls retrieval of Na^+^ from the xylem in Arabidopsis. Plant Cell Environ..

[59] Berthomieu P., Conejero G., Nublat A., Brackenbury W.J., Lambert C., Savio C., Uozumi N., Oiki S., Yamada K., Cellier F. (2003). Functional analysis of *AtHKT1* in Arabidopsis shows that Na 1 recirculation by the phloem is crucial for salt tolerance. EMBO J..

[60] Senadheera P., Singh R., Maathuis F.J. (2009). Differentially expressed membrane transporters in rice roots may contribute to cultivar dependent salt tolerance. J. Exp. Bot..

[61] Nayyar H., Kaur R., Kaur S., Singh R. (2014). γ-Aminobutyric acid (GABA) imparts partial protection from heat stress injury to rice seedlings by improving leaf turgor and upregulating osmoprotectants and antioxidants. J. Plant Growth Regul..

[62] Vijayakumari K., Puthur J.T. (2015). γ-Aminobutyric acid (GABA) priming enhances the osmotic stress tolerance in *Piper nigrum* linn. plants subjected to PEG-induced stress. Plant Growth Regul..

[63] Krishnan S., Laskowski K., Shukla V., Merewitz E.B. (2013). Mitigation of drought stress damage by exogenous application of a non-protein amino acid gamma aminobutyric acid on perennial ryegrass. J. Am. Soc. Hortic. Sci..

[64] Mittler R. (2002). Oxidative stress, antioxidants and stress tolerance. Trends Plant Sci..

[65] Sharma P., Jha A.B., Dubey R.S., Pessarakli M. (2012). Reactive oxygen species, oxidative damage, and antioxidative defense mechanism in plants under stressful conditions. J. Bot..

[66] Swigonska S., Amarowicz R., Król A., Mostek A., Badowiec A., Weidner S. (2014). Influence of abiotic stress during soybean germination followed by recovery on the phenolic compounds of radicles and their antioxidant capacity. Acta Soc. Bot. Pol..

[67] Ma Y., Wang P., Chen Z., Gu Z., Yang R. (2018). GABA enhances physio-biochemical metabolism and antioxidant capacity of germinated hulless barley under NaCl stress. J. Plant Physiol..

[68] Kovacik J., Klejdus B., Hedbavny J., Backor M. (2009). Salicylic acid alleviates NaCl-induced changes in the metabolism of Matricaria chamomilla plants. Ecotoxicology.

[69] Kim S.K., Son T.K., Park S.Y., Lee I.J., Lee B.H., Kim H.Y., Lee S.C. (2007). Influences of gibberellin and auxin on endogenous plant hormone and starch mobilization during rice seed germination under salt stress. J. Environ. Biol..

[70] Dai H.X., Cao F.B., Chen X., Zhang M., Ahmed I.M., Chen Z.H., Li C., Zhang G.P., Wu F.B. (2013). Comparative proteomic analysis of aluminum tolerance in Tibetan wild and cultivated barleys. PLoS ONE.

[71] Ahammed G.J., Choudhary S.P., Chen S., Xia X., Shi K., Zhou Y. (2013). (2013) Role of brassinosterods in alleviation of phenanthrene-cadmium co-contamination-induced photosynthetic inhibition and oxidative stress in tomato. J. Exp. Bot..

[72] Thipyapong P., Hunt M.D., Steffens J.C. (2004). Antisense down regulation of polyphenol oxidase results in enhanced disease susceptibility. Planta.

[73] Hong J.K., Hwang B.K. (2002). Induction by pathogen, salt and drought of a basic class II chitinase mRNA and its in situ localization in pepper (*Capsicum annuum*). Physiol. Plant..

[74] Yeh S., Moffatt B., Griffith A., Xiong M., Yang F., Wiseman D.S., Sarhan S.B., Danyluk F.J., Xue Y.Q., Hew C.L. (2000). Chitinase genes responsive to cold Encode antifreeze proteins in winter cereals. Plant Physiol..

[75] Xiang Y., Huang Y., Xiong L. (2007). Characterization of stress-responsive CIPK genes in rice for stress tolerance improvement. Plant Physiol..

[76] Seki M., Narusaka M., Ishida J., Nanjo T., Fujita M., Oono Y., Kamiya A., Nakajima M., Enju A., Sakurai T. (2002). Monitoring the expression profiles of 7000 Arabidopsis genes under drought, cold and high-salinity stresses using a full-length cDNA microarray. Plant J..

[77] Xiong L., Yang Y. (2003). Disease resistance and abiotic stress tolerance in rice are inversely modulated by an abscisic acid-inducible mitogen-activated protein kinase. Plant Cell.

[78] Anderson J.P., Badruzsaufari E., Schank P.M., Manners J.M., Desmond O.J., Ehlert C., Maclean D.J., Ebert P.R., Kazan K. (2004). Antagonistic interaction between abscisic acid and jasmonate-ethylene signaling pathways modulates defense gene expression and disease resistance in Arabidopsis. Plant Cell.

[79] Bowler C., Fluhr R. (2000). The role of calcium and activated oxygen as signals for controlling cross-tolerance. Trends Plant Sci..

[80] West G., Inze D., Beemster G.T. (2004). Cell cycle modulation in the response of the primary root of Arabidopsis to salt stress. Plant Physiol..

[81] Zhao L., Wang P., Hou H., Zhang H., Wang Y., Yan S., Huang Y., Li H., Tan J., Hu A. (2014). Transcriptional regulation of cell cycle genes in response to abiotic stresses correlates with dynamic changes in histone modifications in maize. PLoS ONE.

[82] Sheteiwy M.S., An J., Yin M., Jia X., Guan Y., He F., Hu J. (2018). Cold plasma treatment and exogenous salicylic acid priming enhances salinity tolerance of *Oryza sativa* seedlings. Protoplasma.

[83] Sheteiwy M.S., Shen H., Xu J., Guan Y., Song W., Hu J. (2017). Seed polyamines metabolism induced by seed priming with Spermidine and 5-aminolevulinic acid for chilling tolerance improvement in rice (*Oryza sativa* L.) seedlings. Environ. Exp Bot..

[84] Li Z., Xu J., Gao Y., Wang C., Guo G., Luo Y., Huang Y., Hu W., Sheteiwy M.S., Guan Y. (2017). The Synergistic priming effect of exogenous salicylic acid and H2O2 on chilling tolerance enhancement during maize (Zea mays L.) seed germination. Front. Plant Sci..

[85] Hu Q., Fu Y., Guan Y., Lin C., Cao D., Hu W., Sheteiwy M.S., Hu J. (2016). Inhibitory effect of chemical combinations on seed germination and pre-harvest sprouting in hybrid rice. Plant Growth Regul..

[86] Hu Q., Lin C., Guan Y., Sheteiwy M.S., Hu W., Hu J. (2017). Inhibitory effect of eugenol on seed germination and pre-harvest sprouting of hybrid rice (*Oryza sativa* L.). Sci. Rep..

[87] Sheteiwy M.S., Gong D., Gao Y., Pan R., Hu J., Guan Y. (2018). Priming with methyl jasmonate alleviates polyethylene glycol-induced osmotic stress in rice seeds by regulating the seed metabolic profile. Environ. Exp. Bot..

[88] Sheteiwy M.S., Guan Y., Cao D., Li J., Nawaz A., Hu Q., Hu W., Ning M., Hu J. (2015). Seed priming with polyethylene glycol regulating the physiological an molecular mechanism in rice (*Oryza sativa* L.) under nano-ZnO stress. Sci. Rep..

[89] Sheteiwy M.S., Fu Y., Hu Q., Nawaz A., Guan Y., Zhan L., Huang Y., Hu J. (2016). Seed priming with polyethylene glycol induces antioxidative defense and metabolic performance of rice under nano-ZnO stress. Environ. Sci. Pollut. Res..

[90] Sheteiwy M.S., Dong Q., An J., Song W., Guan Y., He F., Huang Y., Hu J. (2017). Regulation of ZnO nanoparticles-induced physiological and molecular changes by seed priming with humic acid in *Oryza sativa* seedlings. Plant Growth Regul..

[91] Zhou W.J., Leul M. (1999). Uniconazole-induced tolerance of rape plants to heat stress in relation to changes in hormonal levels, enzyme activities and lipid peroxidation. Plant Growth Regul..

[92] Li H.S. (2000). Principle and Technology of Plant Physiological and Biochemical Experiments.

[93] Velikova V., Yordanov I., Edreva A. (2000). Oxidative stress and some antioxidant systems in acid rain treated bean plants. Plant Sci..

[94] Jiang M., Zhang J. (2001). Effect of abscisic acid on reactive oxygen species, antioxidative defence system and oxidative damage in leaves of maize seedlings. Plant Cell Physiol..

[95] Zheng H.Z., Cui C.L., Zhang Y.T., Wang D., Jing Y.K., Kim Y. (2005). Active changes of lignification-related enzymes in pepper response to Glomus intraradices and/or Phytophthora capsici. J. Zhejiang Univ. Sci. B.

[96] Wyrambik D., Grisebach H. (1975). Purification and properties of isoenzymes of cinnamyl-alcohol dehydrogenase from soybean cell-suspension cultures. Eur. J. Biochem..

[97] Zhang C.H., Ying G.E. (2008). Response of Glutathione and Glutathione S-transferase in Rice Seedlings Exposed to Cadmium Stress. Rice Sci..

[98] Zhao X., Wang W., Zhang F., Deng J., Li Z., Fu B. (2014). Comparative metabolite profiling of two rice genotypes with contrasting salt stress tolerance at the seedling stage. PLoS ONE.

[99] Hu Y., Zhang L., Zhao L., Li J., He S., Zhou K., Yang F., Huang M., Jiang L., Li L. (2011). Trichostatin A selectively suppresses the cold-induced transcription of the *ZmDREB1* gene in maize. PLoS ONE.

