# Supplementary material for: GABA-Alleviated Oxidative Injury Induced by Salinity, Osmotic Stress and their Combination by Regulating Cellular and Molecular Signals in Rice
Source: Int J Mol Sci. 2019 Nov 14;20(22):5709. doi: 10.3390/ijms20225709 (PMC6888568; doi:10.3390/ijms20225709)
Supplement: Supplementary file 1 [file ijms-20-05709-s001.pdf]

Supplementary Table S1. Sequences of oligonucleotide primers used in QRT-PCR.

Primer name	Primer orientation	Sequence (5'-3')
ACT	Forward	CTTCATAGGAATGGAAGCTGCGGGT
	Reverse	CGACCACCTTGATCTTCATGCTGCT
PAL1	Forward	ATAGAGCGGGAGGTCAACTC
	Reverse	AAGGCAGACCGTTGTTGTAG
PPO	Forward	TCTACAACGAGAGGCGTGAC
	Reverse	GGTCGATCAGCTGGTCTCTT
IbCAD1	Forward	ATCTTGATTGTCTCAATCTA
	Reverse	GGACATTATTACATTACAC
SKDH	Forward	TCTTGCAAACACAACAGCAA
	Reverse	CACATTCTGCAGCTTCCCTA
SbGST	Forward	CTCGAGAGGTGAAAGTTCTAGGA
	Reverse	GGTACAGCTGCTTGGTACTTAGCCA
Chi2	Forward	GCTAGGTTGGCATGATCAAA
	Reverse	GAACGCAACTCTCTCCCTTC
APXa	Forward	GCTGAGTGACCCTGCCTTCC
	Reverse	CCCCAGTTCGGAGAGCTTGA
CATa	Forward	GGAAGCTGTTCGTCCAGGTGAT
	Reverse	TCCGGCCATGTCTTGGTGTC
SOD1	Forward	GGGAGATGGTCCCACCACTG
	Reverse	GGCCCAGTTGACATGCAACC
OsCIPK01	Forward	CATGAAAAATGGCAGGGGTT
	Reverse	CAGACGGGCAAGAAGAGAGC
OsCIPK02	Forward	ATGGGCATTGATGCAGAGAT
	Reverse	GCAAAAACAGTAACATCCAGAAAC
OsCIPK03	Forward	CAGGCACTGAATCTGGACAA
	Reverse	GCTACTCTACGGCGAACACC
OsCIPK07	Forward	ATGGAGATGTCGGAGGTGTC
	Reverse	CATTCCTACCAACAATTTAG
OsCIPK08	Forward	TGAGGCATCCGAATGTGGTT
	Reverse	CGCCAGTGATGAACTCCAAGAT
OsCIPK09	Forward	CTCGACCGCAACCATGTG
	Reverse	TCATTGTGGAAATCTCCCGTTT
OsCIPK12	Forward	TTTACCGGACCAAACCTGAC
	Reverse	AATGGCATCAGGAATGGAAG
OsCIPK15	Forward	AGGAGGGAAGGAATGGTGTT
	Reverse	TTGAAAGGCTCAAAGCCAAT
OsCIPK17	Forward	GCTTGGCCCTTCTGTAAATG
	Reverse	CGGTGGCTAGTTTTTGAACC
